# Exploring the history of an unsung hero in pioneering biomedical research: Dr Yellapragada Subbarow

**DOI:** 10.1097/MS9.0000000000003621

**Published:** 2025-07-22

**Authors:** L V Simhachalam Kutikuppala, Sushil Sharma, C Madhavrao, Gaurav Rangari, Arup Kumar Misra, Srinivasa Rao Katiboina, Pavani Saggurthi, Golla Varshitha

**Affiliations:** a Department of Pharmacology, All India Institute of Medical Sciences (AIIMS), Mangalagiri, Andhra Pradesh, India; b Department of Internal Medicine, International School of Medicine (ISM), Bishkek, Kyrgyzstan

## Introduction and background

**Figure 1.**
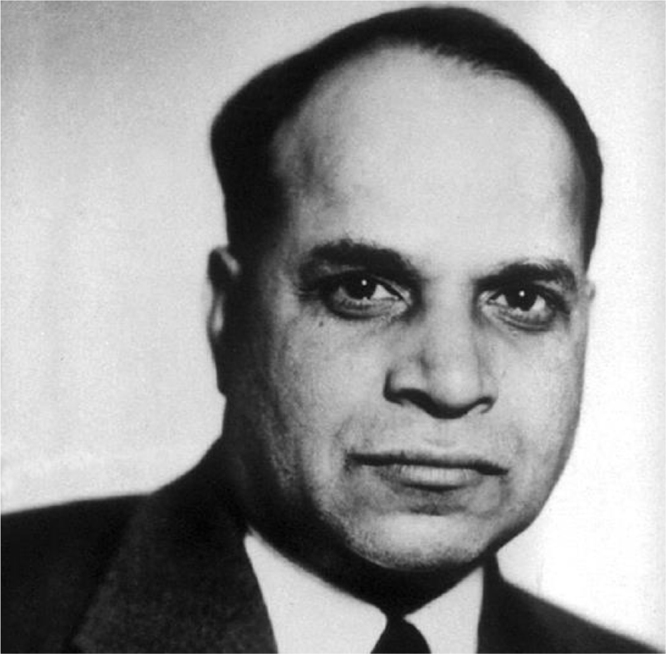
Photograph of Dr Yellapragada Subbarow^[3]^.

Born on 12 January 1895 in a remote Indian village called Bhimavaram, Dr Yellapragada Subbarow is an unsung scientist and pioneer of biomedical research. Even though he is still not well-known, his revolutionary discoveries in biochemistry and medicine have had a significant impact on human health^[1]^ (Fig. 1). The person who made the discovery of an important and most used anticancer molecule, the methotrexate is credited with some of the greatest contributions to many fundamental scientific fields, including biochemistry, microbiology, pharmacology, oncology, and other allied sciences^[2]^. These contributions are still being used in many experiments that are important to science today. Most global medical professionals are still unaware of his work, “The colorimetric determination of phosphorus,” which was published in the Journal of Biological Chemistry and is one of the most cited works worldwide with over 21 000 citations^[3]^. During his early phases of career, he enrolled in Madras Medical College in Chennai, India, to further drive his passion in medicine. Due to his involvement in the Indian independence struggle, he was not granted the full Bachelor of Medicine and Bachelor of Surgery (MBBS) degree, but rather was given a lower Licentiate of Medicine and Surgery certificate. He did not hold an MBBS degree; thus, he began his career at Madras Ayurvedic College as an anatomy and physiology instructor and worked his way up to vice principal. During an accidental encounter with American physician John Fox Kendrick, the ambition to attend Harvard School of Tropical Medicine was planted^[4]^.

## Review

### Challenges in the early career

Due to the early deaths of close relatives from illness, he experienced a painful time during his time in school. Eventually, he matriculated from the Hindu High School, Chennai, on his third attempt. Dr Subbarow was able to enter the Harvard School of Tropical Medicine by overcoming financial obstacles. There, his inferior license prevented him from being eligible for an internship or scholarship, so he had to work part-time to make ends meet^[3,5]^. Dr Subbarow was not offered a full-time position at Harvard due institutional and racial biases prevalent at the time. Despite his groundbreaking work, Subbarow faced discrimination because of his ethnicity, which hindered his professional advancement in Western academic institutions like Harvard. Furthermore, there were concerns regarding his unconventional approach to scientific research and the lack of formal academic credentials. His situation underscores the challenges faced by non-Western scientists in securing positions in prestigious institutions^[6]^.

### His contribution to modern medicine

After earning his degree from the Harvard School of Tropical Medicine, he enrolled in Harvard Medical School in 1924. Together with Dr Cyrus Fiske, he created the Fiske–Subbarow method at Harvard Medical School, which is one of the most often mentioned studies in the scientific literature for phosphorus quantification in bodily fluids^[1,2]^.

Over the years, Subbarow became close with Fiske and felt a feeling of loyalty to his mentor and advisor, something he never regretted. He gained recognition in biochemistry textbooks for his findings regarding the function of adenosine triphosphate (ATP) and phosphocreatine in muscle activity. His research and writings on the Fiske–Subbarow phosphorus estimating method paid off, as he was granted his Ph.D. at the Harvard Commencement Program in 1929^[2,4]^.

**Figure 2.**
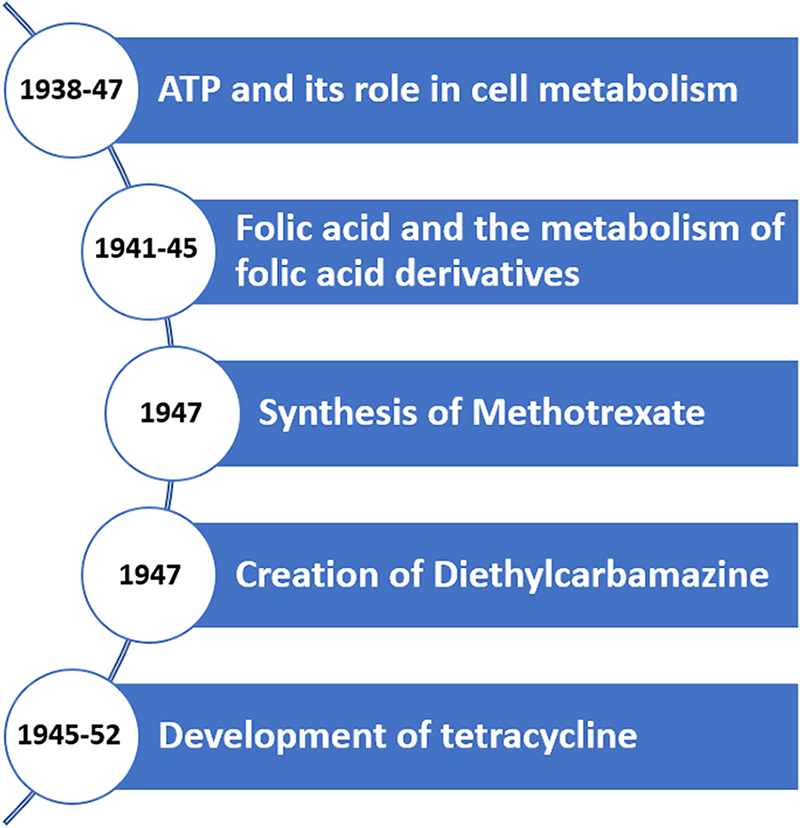
Key contributions (year-wise) of Dr Yellapragada Subbarow.

A folic acid analogue, which was actually a folic acid antagonist, was taken into consideration by Mount Sinai Hospital researchers in the 1940s as a potential cancer treatment since it inhibited tumor cells in mice. Following up on this research, Sidney Farber of Boston Children’s Hospital gave folic acid to kids with leukemia as part of a clinical trial. To his dismay, though, he saw an acceleration in the growth of tumor cells. After drawing the conclusion that folic acid antagonists might prevent tumor growth, Farber convinced Subbarow and associates to create a folic acid antagonist. Aminopterin, an antifolate drug that has been demonstrated to be useful in treating paediatric leukemia, was synthesized by Subbarow’s group^[2,3]^. Both aminopterin and methotrexate are folate antagonists belonging to a same class of medications called antimetabolites. Aminopterin is a precursor to methotrexate in terms of the chemical structure, whereas methotrexate is a modified form of aminopterin^[3]^ (Fig. 2).

In 1940, after Harvard had refused to give him a full position, Subbarow joined Lederle Laboratories and began working on creating new medications. Important research findings from his laboratory included the extraction and manufacture of folic acid and vitamin B12, as well as the identification of the tetracycline class of antibiotics and diethylcarbamazine, both of which were later applied in clinical settings. Third-generation tetracycline doxycycline is used as a prophylactic against malaria and was essential in treating the plague. Worldwide, diethylcarbamazine is used to eradicate filariasis. In 1941, he brought retired mycology expert Prof. Benjamin Duggar into his group to advise on mycology research aimed at finding novel antibiotics. At an event hosted by the New York Academy of Sciences, Duggar presented aureomycin, the first tetracycline antibiotic in history, and Subbarow was relegated to the sidelines in the Roosevelt Memorial. As the laboratory’s director of research, he continued to work there until his passing in 1948^[1,5]^.

### His participation in the achievements obtained

Dr Yellapragada Subbarow made groundbreaking contributions to biochemistry, particularly in the fields of nucleic acid synthesis and energy metabolism. His work on the enzymatic synthesis of ATP was instrumental in understanding the cellular energy transfer^[6]^. He also interpreted the structure of folic acid and its essential and vital role in cell metabolism, that had significantly advanced biochemistry of vitamins^[7]^. Moreover, his collaboration with Harington led to the isolation and delineation of a coenzyme of folic acid, advancing the knowledge of folate metabolism^[8]^. Subbarow’s exploration of the phosphates role in biological systems shed light on their significance in cellular processes^[9]^, while his study of role of adenosine in synthesis of nucleic acid contributed to better understanding of DNA and RNA synthesis^[10,11]^. These achievements were crucial to the development of molecular biology and modern biochemistry.

## Conclusion

During his lifetime, Dr Subbarow’s significant contributions allowed him to be included in biochemistry textbooks, but the scientific community largely ignored his accomplishments, making him an unsung hero of clinical medicine. The most illuminating aspect of his life was the quantum of energy and enthusiasm he gave selflessly to scientific research and his devotion to medicine; his efforts always seemed to be more focused on research than getting credit for it, and his modest nature of sharing credit with his junior researchers, who worked assiduously under his guidance, was indeed a rare quality.

## Data Availability

N/A.
